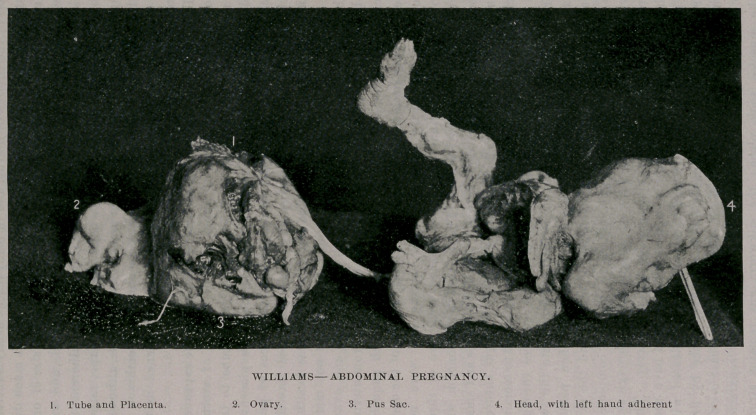# Report of a Recent Case of Abdominal Pregnancy, Complicated with Pyosalpinx,—Laparatomy, Recovery

**Published:** 1895-02

**Authors:** H. T. Williams

**Affiliations:** 19 Clinton Place; Rochester, N. Y.


### REPORT OF A RECENT CASE OF ABDOMINAL PREGNANCY, COMPLICATED WITH PYO-SALPINX—LAPARATOMY, RECOVERY.

By H. T. WILLIAMS, M. D., of Rochester, N. Y.

THE patient was referred to me by Dr. J. H. Finnessy, of Rochester, N. Y., in whose practice it occurred, and was admitted to St. Mary's hospital September 10, 1894, with the following history:

Mrs. Geo. L., born in Canada, 29 years of age; married nine years; one child, now 8 years of age.

*Menses* irregular ever since birth of child. Menses ceased last December; some morning sickness followed; she increased in flesh. This increase was more noticeable after the sixth month. The abdomen increased in size until fore part of last April, when she had a sudden flow of water from her vagina which continued all of one day. Since that time her abdomen has gradually diminished in size; but ever since the flow of water from the vagina, she had had occasional sharp, shooting pains through the abdomen. Last July, noticed that her breasts were very large and contained considerable milk for two weeks, when it gradually disappeared and breasts grew smaller until at the time of her admission to hospital they had reduced to their normal size.

In August last, for two days, had severe pains in abdomen, which resembled labor pains; since then she has been comparatively comfortable, with the exception of occasional slight pains. In the early part of August, a few days after the attacks of severe pain, she menstruated normally, the flow lasting four or five days; she menstruated again September 1st, one week before operation.

*Vaginal examination* reveals a tumor somewhat irregular in shape, lying above and to both sides of the uterus, but by far the greater part of it to the right of the womb.

She was operated upon September 10, 1894, about an hour and a half after her admission into the hospital. An incision three and one-half inches in length was made in the median line of the abdomen between the umbilicus and symphysis pubis. The fetus (a male) was found still covered with the amniotic sac (which was empty and firmly adherent to fetus) lying in the abdominal cavity

---

1 Read before the Central New York Medical Association, Buffalo, N. Y., October 16, 1894.

WILLIAMS—ABDOMINAL PREGNANCY.

1. Tube and Placenta. 2. Ovary. 3. Pus Sac. 4. Head, with left hand adherent on top of the fundus of uterus, and in many places firmly adherent to intestines, which, however, were stripped off without much difficulty and the fetus easily removed. It was about the size of one of five months, but had evidently been dead for some time, and was considerably shrunken and macerated. One hand is adherent to top of cranium. The head is somewhat irregular in shape, rather triangular, and apparently there are no eyes, ears or nose, although this is undoubtedly due to the fact that the amniotic membrane is now so firmly adherent to the head and so much thickened as to obliterate them.

The placenta, which is of good size, was found in the expanded Fallopian tube, to the right of upper part of fundus of uterus. The umbilical cord passed through an opening in the tube to the umbilicus of the child. A part of the tube near the placental attachment was sacculated and contained several ounces of pus, which burst during removal, and some of the pus escaped into the abdominal cavity; the ovary on that side (the right) was healthy, but had to be removed with the tube. There were not many troublesome adhesions, and the tube with the placenta and ovary was ligated with silk ligature three-fourths of an inch from uterine attachment and removed, and the stump cauterized with paquelin cautery. Very little bleeding occurred during any part of the operation. The left ovary and tube were healthy and were not removed; the abdominal cavity was flushed with two or three quarts of warm sterilized water. The incision in peritoneum was closed with fine running catgut suture. The aponeuroses and small amount of muscular fibers closed with five silkworm gut sutures, which were tied with two knots, then cut short and allowed to remain; then a few intermediate medium-sized catgut sutures through anterior aponeurosis of muscles to perfectly approximate the two surfaces. Several large-sized catgut sutures were then passed through the skin and down to aponeurosis of muscle and the skin then closed over the silkworm-gut sutures, in this way completely burying them; a few intermediate fine catgut sutures through skin entirely closed incision, approximating the surfaces nicely. This method of closing the incision in laparatomies is one I have used in a number of cases recently, and have been greatly pleased with the results. In all but two cases union has taken place by first intention, and in these two cases (of which the above case is one) only the skin separated for a short distance, but no separation of the muscles took place; the silkworm gut remains strong and unirritating, and it is almost impossible for a hernia to occur.

The after-dressing consists in dusting the wound with a little powdered iodoform; then covered with iodoform and bi-chloride gauze held in place by two strips of adhesive plaster extending across abdomen, then a layer of absorbent cotton and a wide flannel bandage completes the dressing.

The patient made a rapid and uneventful recovery, with very little constitutional disturbance of any kind. The highest temperature, 100 4-5°, occurred on the fourth day after the operation, and dropped to 99° after a free movement of the bowels had taken place.

The incision healed by first intention with the exception of about an inch of the upper part, which separated through skin and fat only, and soon entirely closed again. She was allowed to get out of bed on the twelfth day, and at the end of the third week was able to walk about as she pleased, and was discharged from the hospital cured.

19 Clinton Place.

## Figures and Tables

**Figure f1:**